# The role of selenium intervention in gut microbiota homeostasis and gene function in mice with breast cancer on a high-fat diet

**DOI:** 10.3389/fmicb.2024.1439652

**Published:** 2024-07-31

**Authors:** Yinan Li, Min Liu, Bingtan Kong, Ganlin Zhang, Qing Zhang

**Affiliations:** ^1^Beijing University of Chinese Medicine, Beijing, China; ^2^Department of Oncology, Beijing Hospital of Traditional Chinese Medicine, Capital Medical University, Beijing, China; ^3^School of Traditional Chinese Medicine, Capital Medical University, Beijing, China

**Keywords:** selenium, gut microbiota, genetic functions, breast cancer, high-fat status, metagenomic sequencing

## Abstract

**Objective:**

This study aimed to investigate the effect of selenium on gut microbiota in mice with breast cancer under a high-fat diet.

**Methods:**

A total of 12 female BALB/c mice were randomly divided into two groups: 4 T1 + selenium+ high-fat diet group and 4 T1 + high-fat diet group. Mice were injected with 4 T1 cells on the right 4th mammary fat pad and kept on a high-fat diet. Fecal samples were collected, and DNA was extracted for metagenomic sequencing and bioinformatics analysis. Relevant target genes and pathways were annotated and metabolically analyzed to explore the intervention effect of selenium on breast cancer in the high-fat diet state.

**Results:**

Selenium supplementation in the high-fat diet altered the composition and diversity of gut microbiota in mice with breast cancer. The gut microbial composition was significantly different in the selenium intervention group, with an increased abundance of Proteobacteria, Actinobacteria, and Verrucomicrobia phyla and species such as *Helicobacter ganmani*, *Helicobacter japonicus*, and *Akkermansia muciniphila*, while phyla, such as Bacteroidetes, Firmicutes, Deferribacteres, and Spirochaetes, and species, such as *Prevotella* sp. *MGM2*, *Muribaculum intestinale*, *Lactobacillus murinus*, and *Prevotella* sp. *MGM1,* were decreased. Functional analysis revealed differential expression of genes related to carbohydrate-active enzymes, pathogen–host interactions, cell communication, cell auto-induction, membrane transporters, and virulence factors. Furthermore, 37 COGs and 48 metabolites with rising metabolic potential in the selenium intervention group were predicted.

**Conclusion:**

Selenium alters the homeostasis of gut microbiota in mice with breast cancer on a high-fat diet, affecting their composition, abundance, and associated metabolism. These findings suggest that the mechanism involves interfering with gut microbiota homeostasis, leading to altered synthesis of tumor-associated proteins and fatty acids and inducing tumor cell apoptosis and pyroptosis.

## 1 Introduction

In 2020, female breast cancer (BC) overtook lung cancer as the most common cancer with 2,261,419 new cases (11.7%), and it became the fifth leading cause of cancer mortality worldwide, with 685,000 deaths (Sung et al., 2021). Obesity is a high-risk factor for the development and recurrence and metastasis of BC (Picon-Ruiz et al., 2017), and it is closely related to BC-associated mortality. The underlying mechanism may be related to the secretion of hormones such as estrogen and insulin and the enzymatic activity of proteins such as aromatase (Engin, 2017). Changes in gut microbiota are often found in patients with BC. A case–control study conducting an inquiry into the association between fecal microbiota and BC in postmenopausal women showed that the diversity of fecal microbiota was altered in BC patients, especially for species of *Clostridiaceae*, *Faecalibacterium*, *Ruminococcaceae*, *Dorea*, and *Lachnospiraceae* (Goedert et al., 2015). Gut microbiota is closely linked to the onset and prognosis of BC, can affect the occurrence, development, and metastasis of this disease through a variety of mechanisms, and drives epithelial cell transformation by affecting genomic stability, impeding apoptosis, and releasing cell proliferation signals. Gut microbiota also plays an important role in host oncogenic pathways versus chemoresistance. Class alterations in gut microbial populations affect hormone synthesis and may lead to higher estrogen levels; therefore, it would increase BC risk, and since the populations of some bacterial types in the gut increase while others decrease, the diversity of the gut microbiota is significantly affected and may be related to the associated catabolism. This may affect estrogen release from the enterohepatic circulation, leading to increased systemic estrogen levels (Feng et al., 2021). Therefore, gut microbiota homeostasis restoration may become a novel therapy for BC.

Selenium, an essential trace element, plays a major role in biological growth and development since it has antioxidant, anti-inflammatory, and immune functions; furthermore, it is involved in the metabolism of thyroid hormones. Selenium interaction with gut microbiota is very complex; furthermore, gut microbiota influences the absorption of selenium. The study by Knezevic et al. (2020) found that gut microbiota may affect selenium absorption through the strong thyroid–gut axis, and, in turn, selenium intake affects gut microbiota through the release of hormones.

Studies have found that selenium intake can change the composition of the gut microbiota to some extent and significantly affect the associated function and metabolism; for instance, the proportion of Bacteroidetes and Proteobacteria was significantly different between the high- and low-selenium areas (Zhang et al., 2021). At present, multiple experiments have shown that selenium can reduce the risk of cancer, such as breast, bowel, prostate, and lung cancer, while modulating the immune system among many other processes (Hatfield et al., 2014; Vinceti et al., 2018). Demircan et al. (2021) investigated the association between pre-diagnostic selenium intake and BC prognosis in a prospective cohort study and showed that pre-diagnostic selenium levels were strongly associated with low mortality and recurrence of invasive BC. Therefore, it is important to explore alterations in gut microbiota in high-fat diet BC-bearing mice after intervention with organic selenium and clarify the relationship between organic selenium and gut microbiota in BC patients. However, the relationship between the gut microbiota and organic selenium in BC mice has rarely been investigated using metagenomic sequencing analysis. This study aimed to further investigate the relationship between organic selenium and gut microbiota composition. To this end, metagenomic sequencing was used to analyze the characteristics and composition of gut microbiota in fecal samples from breast cancer mice fed a high-fat diet after organic selenium intervention. Based on the composition of gut microbiota in BC mice fed a high-fat diet after organic selenium intervention, gene function and related metabolites in breast cancer patients were predicted.

## 2 Materials and methods

### 2.1 Animals used in experiments

The current investigation involved female BALB/c mice that were 8 weeks old (Beijing HFK Bioscience Co., Ltd., Beijing, China). The animal study approval number for this study is VS212601356. In accordance with standard laboratory procedures, the animal models were established in Beijing Viewsolid Biotechnology Co., Ltd., and all animals were housed at the Beijing Hospital of Traditional Chinese Medicine (22–24°C, 40–60% relative humidity), food and water were available *ad libitum*, and the light/dark cycle was maintained for 12/12 h, with the light turned on at 6:00 am. The selenium-enriched microalgal protein contained Se (VI): N. D., Se (IV): 14.975, SeCs2: 173.433, MeSeCys: 14.468, SeMet: 5.104, and GSSeGS: 18.249 (μg/g) and was provided by Enshi Zaoyuan Selenopeptide Biotechnology Co. Ltd.

### 2.2 Selenium therapy in an animal model

The cell line 4 T1 is a breast tumor cell line originating from spontaneous breast tumors in BALB/c mice. These highly invasive and tumorigenic cells behave very similarly to human breast cancer in terms of growth, metastasis, and diffusion. Female BALB/c mice were divided into two groups (*n* = 6 in each group) according to their body weight, as shown below: (I) 4 T1 + high-fat diet group was fed with a high-fat diet and distilled water of 0.4 mg/kg via intragastric injection administration (IG) daily (control group) and (II) 4 T1 + selenium + high-fat diet group was fed with a high-fat diet and selenium-enriched microalgal protein of 0.4 mg/kg via IG daily (selenium group). All mice were subcutaneously xenografted with 1 × 10^5^ 4 T1 cells/50 μL each in the right fourth mammary fat pad under anesthesia. The observation period began with the injection of 4 T1 cells for 4 weeks. The high-fat diet consists of 45% kcal fat, 35% kcal carbohydrates, and 20% kcal proteins. The high-fat diet composition is as follows: 10% sucrose, 15% lard, 2% cholesterol, 0.2% sodium cholate, and 72.8% basic feed, provided by Beijing Viewsolid Biotechnology Co., Ltd.

The selenium-enriched microalgal protein was dissolved in deionized water to develop a product with a stock solution that could be used to prepare treatment media for intragastric injection administration. The volume of the IG solution was given at 10 mL/kg according to the mice’s body weight.

### 2.3 Sample collection and DNA extraction

Stool samples were collected in a specimen collection kit and stored at −20°C immediately after defecation and then at −80°C before further manipulation in the laboratory. DNA was extracted from samples using the stool DNA extraction Mini Kit (DSN362, ONREW, Foshan, China). Then, 0.2 g of fecal sample was added to Glass Beads Tube I, after which 600 μL of Buffer STL and 20 μL of Proteinase K were added. Samples were vortexed at 13,000 *g* for 5 min and further lysed by heating at 70°C for 15 min; after the samples had returned to room temperature, 200 μL of Buffer IRP was added to the samples, which were vortexed thoroughly to mix. After placing the sample on ice for 5 minutes, centrifuge it at 13000 g for 3 minutes, take 450 μ L of the resulting supernatant and place it in a 2 mL centrifuge tube. Add 450 μ L of buffer MBL to the centrifuge tube, invert the tube 3-4 times, vortex for 15 seconds, and then let it stand at 55°C for 10 minutes. During this period, mix several times by inversion, add 450 µ L of anhydrous ethanol to the centrifuge tube, vortex for 15 seconds, and briefly centrifuge. The extracted supernatant was purified following the instructions of DNA Extraction Mini Columns II to obtain sample DNA and stored at −20°C. DNA was fragmented to a fragment size of 200–300 bp for library construction, paired-end sequencing was performed using PE150, and metagenomics was performed on the Illumina HiSeq platform following the manufacturer’s instructions (sequencing instrument model: NovaSeq6000).

## 3 Metagenomic sequencing

### 3.1 Quality control of integrated data

All raw metagenomic sequencing data were quality-controlled by MOCAT2 software (Kultima et al., 2016). All the original sequencing reads were disjointed by Cutadapt software (Kechin et al., 2017) and were trimmed by the SolexaQA package with a quality of less than 20 and a length of less than 30 bp (Cox et al., 2010). Clean reads were obtained by quality control. We used SOAPaligner to compare the filtered reads with host reads whose genome was decontaminated to obtain high-quality clean data (Li et al., 2009).

### 3.2 Statistical method

The clean reads were used to be the input data, and we used MetaPhlAn3 (Segata et al., 2012) to make a Classification of species; thus, the relative abundance of the sample bacteria from the species level to the phylum level was obtained. Based on the species annotation results of MetaPhlAn3, we calculated these indicators using the diversity function in the Vegan package of the R programming language. The clean reads obtained from quality control were assembled by *De Novo* using SOAPdenovo to obtain scaftigs with a length greater than 500 bp: reads were interrupted into K-mer to construct the de Bruijn graph, and the Eulerian path was searched to assemble into contigs. Then, scaffolds were connected according to the location relationship of pin-end reads, and we selected successive contigs from scaffolds to get scaftigs. Among them, the k-mer of each sample was calculated by MOCAT2 according to the length and quantity of sample reads. Based on scaftigs obtained by the assembly, we used Meta Gene Mark for gene structure prediction, and CD-HIT was used for clustering to eliminate redundancy after the gene set was obtained (Fu et al., 2012). If the sequence consistency of the two genes is greater than 95%, and the overlap area covers more than 90% of the short sequences, then the two genes will be clustered into a cluster. The longest sequence in the cluster was selected as the representative sequence of the cluster to construct a non-redundant gene set with a gene length greater than 100 bp. Finally, we compared high-quality reads to the constructed non-redundant reference gene set by BWA, and the reads with length less than 30 bp and consistency less than 95% were removed to obtain the reads count of each gene. We downsized count data by using the rrarefy function in the vegan package of R language to obtain the corrected gene abundance.

### 3.3 Sequencing data analysis

#### 3.3.1 Microbial community structure and diversity analysis

The species accumulation curve describes how species increase as the sample size increases condition, which can be used to determine the adequacy of sample size and estimate species richness. (Simpson et al., 2017). Alpha diversity includes richness, evenness, and diversity Shannon index, which can be used to describe genes and functions in samples and the diversity of species. Principal component analysis (PCA) is a simplified analysis of data that effectively finds the most “dominant” elements and structures in the data. Principal coordinate analysis (PCoA) is a visualization method to study data similarity or difference, which is similar to PCA analysis. The difference is that PCoA uses the Bray–Curtis distance. Permutational multivariate analysis of variance (PERMANOVA) was used to assess whether there were significant community differences among different groups. Non-metric multidimensional scale analysis (NMDS) is a data analysis method that can simplify the study objects (samples or variables) in a multidimensional space to a low-dimensional space for positioning, analysis, and classification, while preserving the original relationships between objects.

Enterotype is a very effective method to distinguish gut microbes, and we divided the clusters according to the composition of microbial communities.

#### 3.3.2 Gene functional analysis

We annotate the data to comprehensive antibiotic resistance database (CARD) (Alcock et al., 2020), Evolutionary genealogy of genes: Non-supervised Orthologous Groups (Egg-NOG) (Huerta-Cepas et al., 2019), carbohydrate-active enzymes database (CAZy), Microbial Viral Resistance Database (MvirDB) (Buchfink et al., 2015), pathogen–host interaction database (PHI) (Urban et al., 2017), QS database (Barriuso and Martinez, 2018), transporter classification database (TCDB) (Saier et al., 2016), and virulence factor database (VFDB) (Chen et al., 2016) to analyze the differences between groups.

#### 3.3.3 DeSeq2 and LEfSe

We used a negative binomial distribution model for differential analysis to search for genes with significant expression changes between groups by DESeq2. LEfSe analysis was used to compare the abundance of biomarkers between two or more groups to find biomarkers with significant differences between groups. Finally, linear discriminant analysis (LDA) was used to reduce the dimensionality of the data and assess the impact of significantly different species (i.e., LDA score). We analyzed the relationships between these factors and the microbiota, as well as the functions of the microbiota, using multiple linear association models (MaAsLin) and performed multivariate association analysis with the results of MetaPhlAn3.

#### 3.3.4 KEGG pathway enrichment analysis and metabolic potential analysis

The resulting data were analyzed using the humann_regroup_table merges annotation results, resulting in KEGG KO annotation results. For the relative abundance of KEGG orthology (KO), KEGG pathway enrichment analysis was finally performed using the GAGE package from R/Bioconductor. Finally, we used the predicted relative metabolic turnover (PRMT) method to calculate the community metabolic potential (CMP) after predicting the resulting relative abundances of genes and to estimate the differences in metabolic potential between the different groups (Noecker et al., 2016).

## 4 Results

### 4.1 Microbial diversity

**Figure 1:**
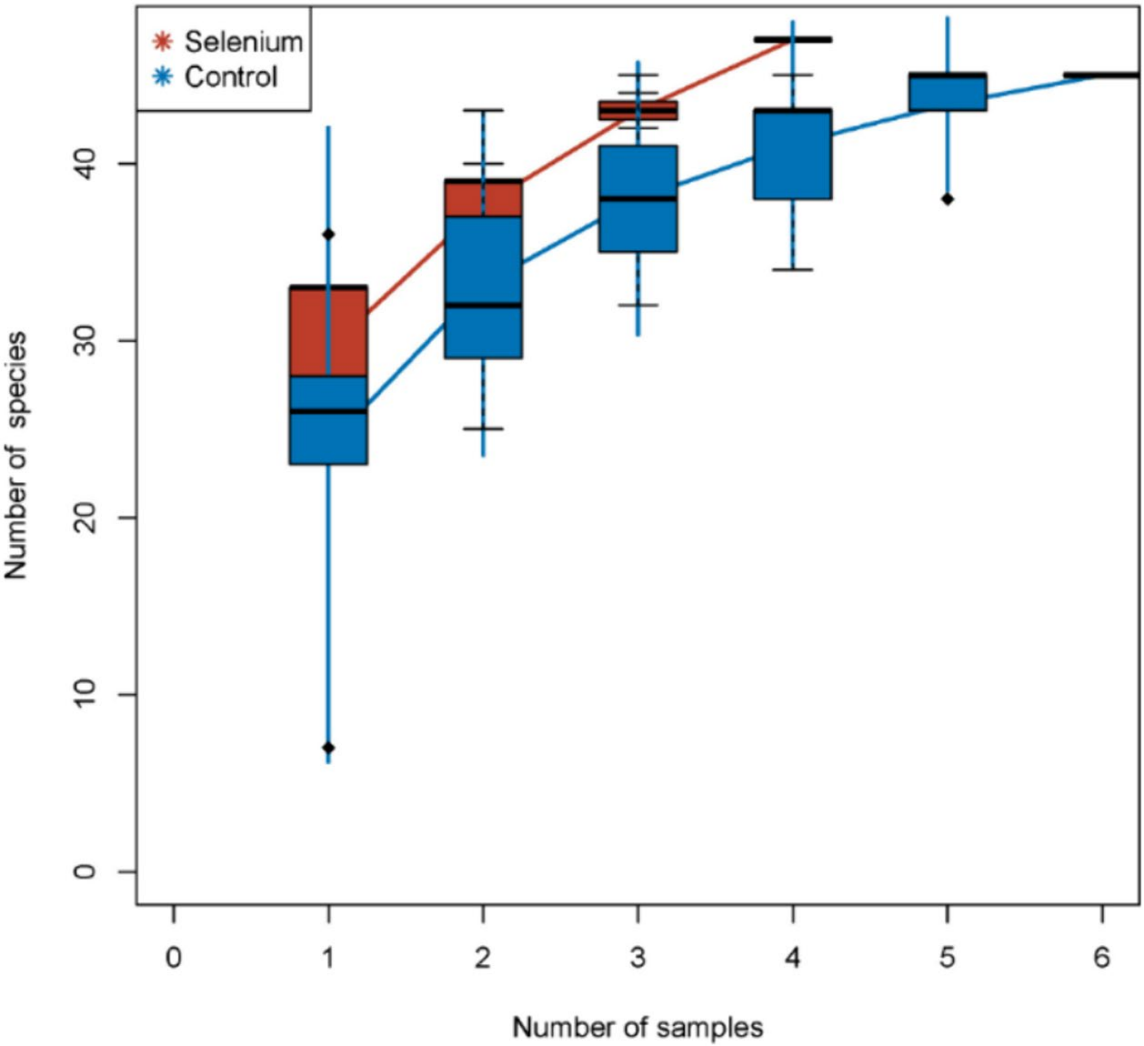
Species accumulation curves. The species accumulation curves of each group level off, indicating that the sample biodiversity is adequately covered by the applied sequencing depth.

**Figure 2:**
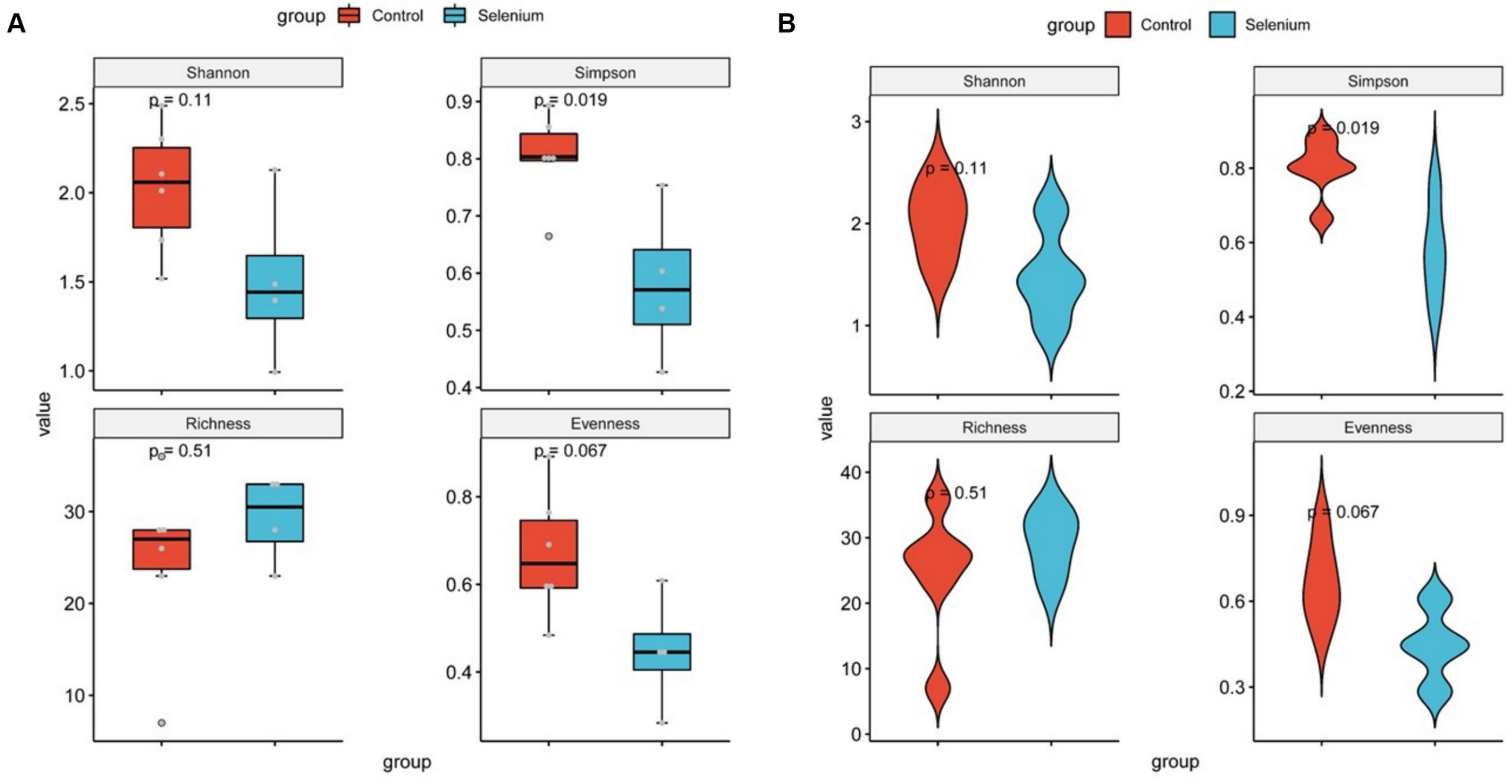
Diversity comparison of different groupings at the gut microbiota level **(A)** and violin plots for comparing diversity in different groupings at the gut microbiota level **(B)**.

**Figure 3:**
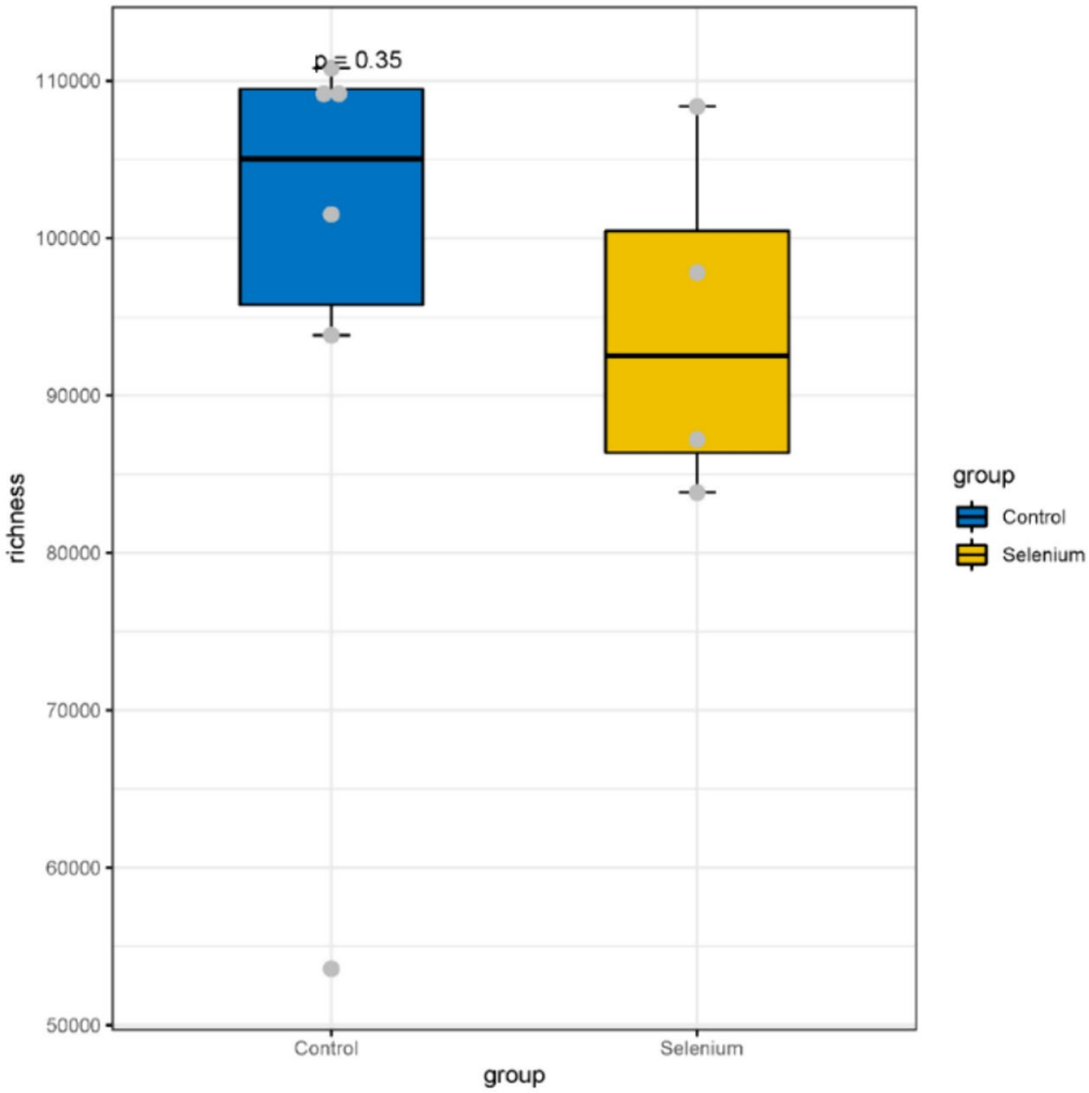
A comparison of the abundances of different groupings at the gene level shows no significant differences between the two groups.

**Figure 4:**
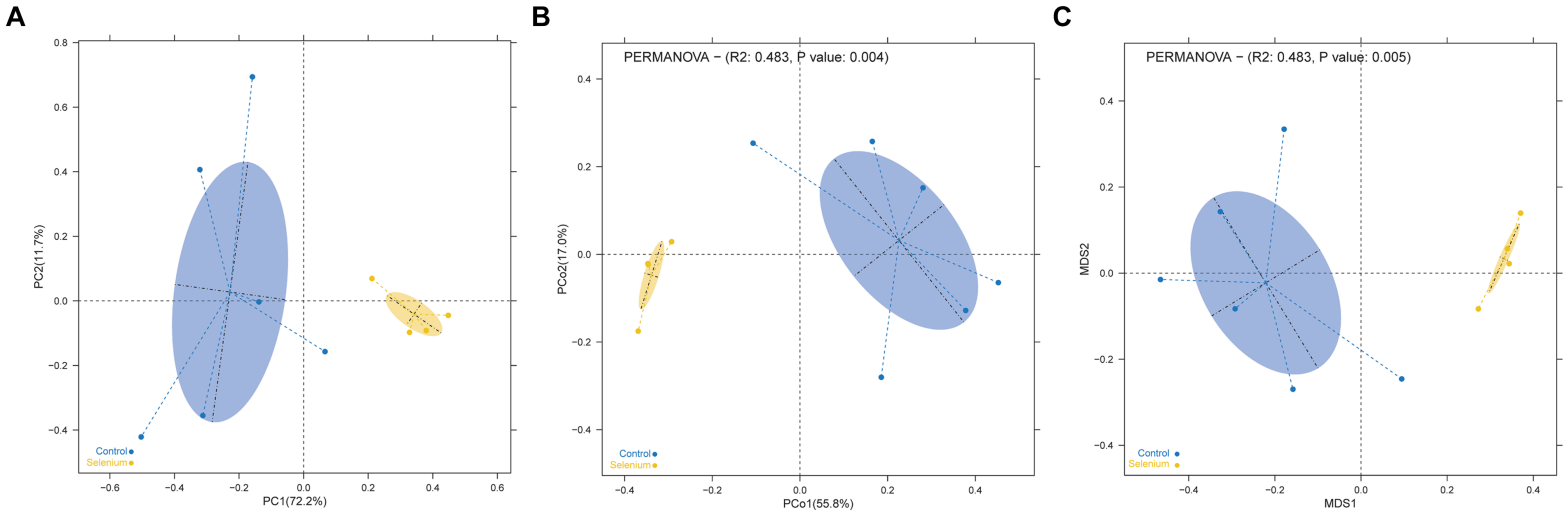
Dimensionality reduction cluster analysis as a two-dimensional PCA **(A)**, PCoA **(B)**, and NMDS **(C)** shows significant differential clustering between the two groups. Yellow dots show the samples from the selenium intervention group, whereas the blue dots show the samples from the control group. The closer the dots in one group, the more similar in gut microbiota. The gut microbiota compositions are indicated with yellow and blue circles, respectively. The smaller the overlap of the two circles, the higher the difference in the gut microbiota compositions between the two groups.

The species accumulation curves of each group leveled off, indicating that the sample biodiversity was adequately covered by the applied sequencing depth (Figure 1). We used Shannon, Simpson, richness, and evenness indexes to represent the alpha diversity of the samples, and we can observe that the Simpson index in the selenium intervention group is significantly lower than that in the control group (*p* < 0.05) (Figure 2). The results of gene richness analysis showed lower values in the selenium intervention group than in the control group, but there was no significant difference between the two groups (Figure 3). This suggests that the intestinal environment of the control group is more suitable for the growth of a certain type of flora and is more uniform. The species richness of gut microbiota increased significantly after selenium intervention. In terms of dimensionality reduction cluster analysis, we performed PCA, PCoA, and NMDS analyses, which revealed significant differences in the composition of gut microbiota between the two groups (Figure 4). In addition, we tested whether the difference between the groups was significantly greater than that within the group using the multiple response permutation procedure, and the differences between the two groups exceeded the differences between samples within each group, indicating that the grouping was homogeneous and that the effects of interventions were significant (Table 1). The smaller the observed-delta value, the smaller the within-group difference, and the larger the expect-delta value illustrates the larger the between-group difference. A-score greater than 0 indicates that the difference between groups is greater than the difference within groups, and A-score less than 0 indicates that the difference between groups is greater than the difference between groups.

**Table 1:** The results of MRPP analysis.

	Group	Distance	A_score	Observe_delta	Expect_delta	P_value	Sig
1	Control_VS_Selenium	Bray–Curtis	0.254	0.254	0.581	0.005	**

### 4.2 Gut microbial alterations

**Figure 5:**
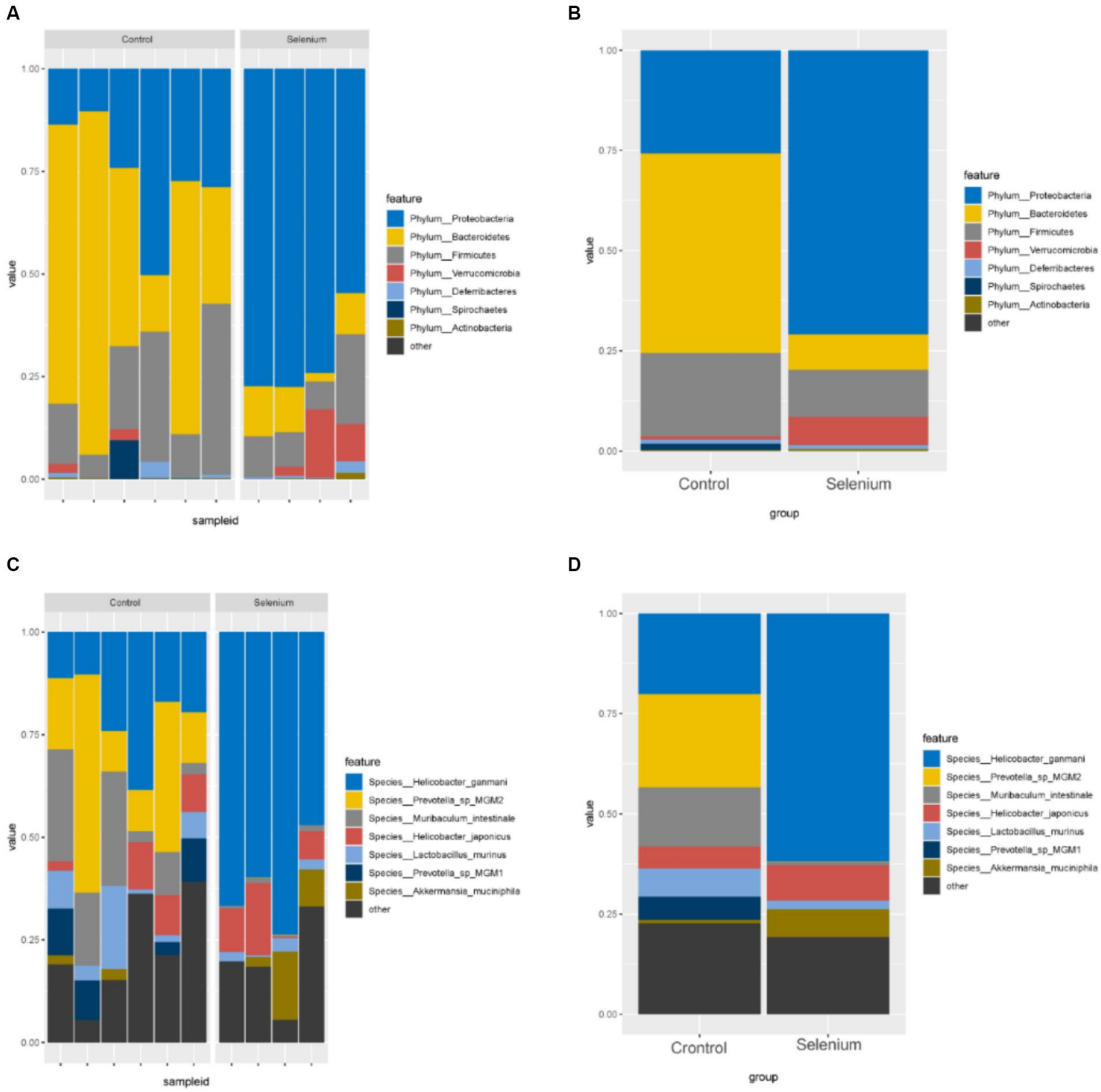
Gut microbiota relative abundance (%) of the different samples **(A)** and different groups **(B)** determined at the phylum, relative abundance (%) of the different samples **(C)** and different groups **(D)** determined at the species.

**Figure 6:**
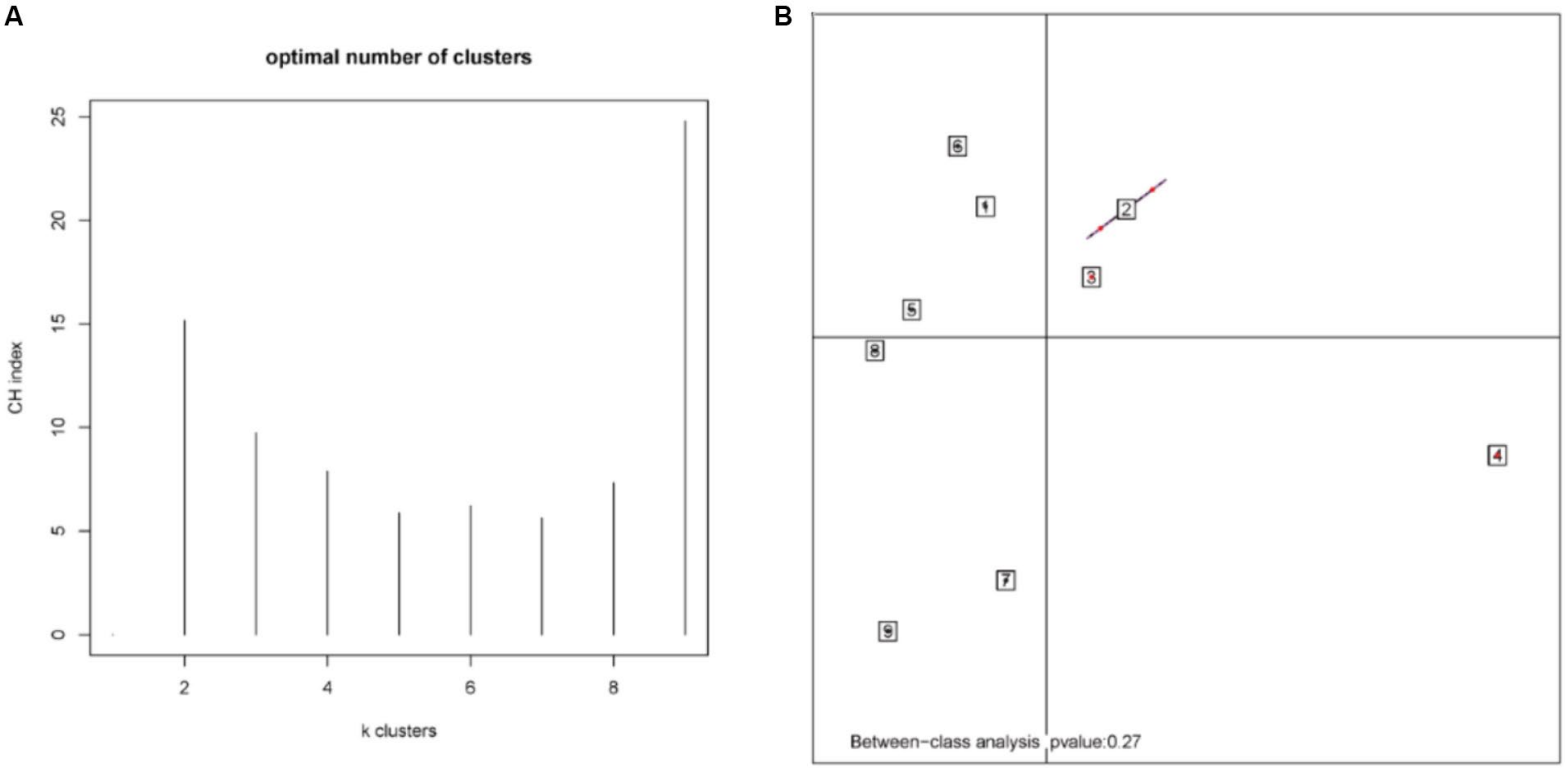
CH index **(A)** and enterotype analysis **(B)**. The different colors represent different clusters. The *p*-value less than 0.05 (derived by the chi-square test) in the plot indicates a significant correlation between enterotype and grouping factors.

According to the different relative abundances in the selenium intervention and control groups, seven dominant phyla were identified. The phyla Bacteroidetes, Firmicutes, Deferribacteres, and Spirochaetes were decreased and Proteobacteria, Actinobacteria, and Verrucomicrobia were increased in the selenium intervention group than in the control group (Figures 5A,B). At the species level, *Helicobacter ganmani, Helicobacter japonicus*, and *Akkermansia muciniphila* were increased, while *Prevotella* sp. *MGM2, Muribaculum intestinale, Lactobacillus murinus*, and *Prevotella* sp. *MGM1* were decreased in the selenium intervention group than in the control group (Figures 5C,D). In addition, we performed enterotype analysis for the two groups of samples, and the best cluster K value was calculated by the Calinski-Harabasz (CH) index (Figure 6A) and then visualized by between-class analysis, which showed no significant difference in enterotype between the two groups (Figure 6B). The Wilcoxon rank-sum permutation test was performed to further compare the significant differences in the composition of gut microbiota between the two groups. The top 20 items with the most significant differences are presented in Table 2.

**Table 2:** Taxonomic differences between the selenium intervention group and the control group (top 20).

	Taxonomy	Control	Selenium	W	*p*-value
1	Species *Parabacteroides goldsteinii*	0	0.282	0	0.006
2	Species *Bacteroides stercorirosoris*	0.004	0.630	0	0.009
3	Phylum Bacteroidetes	49.814	8.821	24	0.010
4	Class Bacteroidia	49.814	8.821	24	0.010
5	Order Bacteroidales	49.814	8.821	24	0.010
6	Family Muribaculaceae	17.558	1.533	24	0.010
7	Genus Muribaculum	14.845	0.920	24	0.010
8	Species Muribaculum intestinale	14.845	0.920	24	0.010
9	Family Prevotellaceae	29.019	0.060	24	0.010
10	Genus Prevotella	29.019	0.060	24	0.010
11	Phylum Proteobacteria	25.797	70.937	0	0.010
12	Class Epsilonproteobacteria	25.717	70.732	0	0.010
13	Order Campylobacterales	25.717	70.732	0	0.010
14	Family Helicobacteraceae	25.717	70.732	0	0.010
15	Genus Helicobacter	25.717	70.732	0	0.010
16	Species *Helicobacter ganmani*	20.157	61.864	0	0.010
17	Species *Parabacteroides gordonii*	0.002	0.189	0	0.011
18	Species Prevotella sp. MGM2	23.214	0.049	24	0.014
19	Family Tannerellaceae	0.145	1.115	1	0.023
20	Genus Parabacteroides	0.145	1.115	1	0.023

W indicates the statistical value of the Wilcoxon rank-sum test. The P. adj indicates the *p*-values corrected by multiple testing.

The selenium intervention group showed a significant increase in the species *Parabacteroides goldsteinii, Bacteroides stercorirosoris, Helicobacter ganmani,* and *Parabacteroides gordonii* and a decrease in *Muribaculum-stercorirosoris* and *Prevotella-sp-MGM2*, compared to the control group. An increase in the genus *Muribaculum* and *Prevotella* and a decrease in the genus *Helicobacter* and *Parabacteroides* were observed in the selenium intervention group. In the selenium intervention group, the families *Helicobacteraceae* and *Tannerellaceae* were more abundant, whereas those of *Muribaculaceae* and *Prevotellaceae* were less abundant. The results of DeSeq2 differential analysis by counting the abundance of species in the two groups showed no significant differences between the groups at the gene level for various species (Table 3).

**Table 3:** Species count data DeSeq2 comparison results between groups (top 5).

	ID	Control	Selenium	baseMean	log2FoldChange	lfcSE	stat	*p*-value
1	Species Abelson murine leukemia virus	1.167	1.750	1.430	0.531	0.900	0.590	0.555
2	Species Abisko virus	1	1	1.030	−0.056	1.062	−0.053	0.958
3	Species *Acanthocystis turfacea* chlorella	1.167	1	1.130	−0.277	1.033	−0.268	0.789
4	Species Acaryochloris marina	1	1	1.030	−0.056	1.062	−0.053	0.958
5	Species Acetoanaerobium sticklandii	4.500	6	5.119	0.386	0.698	0.554	0.580

The results showed no significant differences between the two groups at the gene level across species.

**Figure 7:**
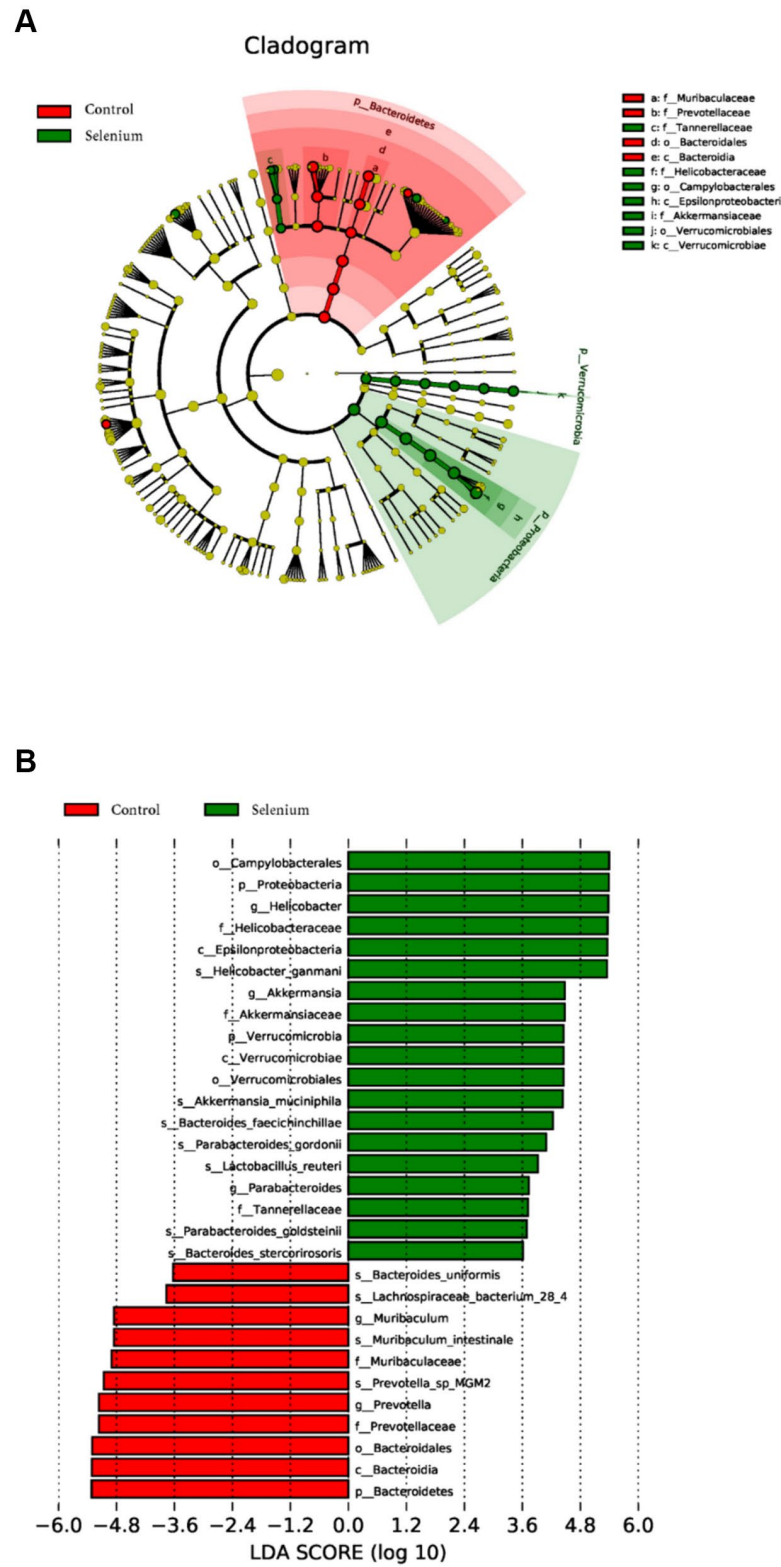
Gut microbiota profiles in the selenium intervention group. The results show cells that differ significantly across taxonomic levels. **(A)** The cladogram generated from the LEfSe analysis indicates the phylogenetic distribution of the microbiota of the selenium intervention group and control group from phylum to genus. **(B)** Histogram of LDA scores to identify differentially abundant bacteria between the selenium intervention group and control group (LDA score > 2.0).

LEfSe was used to further determine the specific significantly different bacterial taxa between the two cohorts (Figure 7A). Several species including *Helicobacter ganmani, Akkermansia muciniphila, Bacteroides faecichinchillae, Bacteroides faecichinchillae, Lactobacillus reuteri, Parabacteroides goldsteinii*, and *Bacteroides stercorirosoris* were significantly enriched in the selenium intervention group. The identified taxa are highlighted on a cladogram to indicate significant differences in phylogenetic distribution and their LDA score (Figure 7B). These results indicated that there was significant gut microbiota alteration between the two groups. MaAsLin results similarly showed that the species *Parabacteroides gordonii, Parabacteroides goldsteinii, Bacteroides stercorirosoris*, and *Lactobacillus reuteri* were increased in the selenium intervention group than in the control group, while *Lachnospiraceae-bacterium-28-4* was decreased (Table 4).

**Table 4:** The results of multivariate linear correlation analysis of gut microbiota indicated that there was significant gut microbiota alteration between the two groups (top 5).

	Feature	Value	Coefficient	*N*	N.not.0	*p*-value
1	Species *Parabacteroides gordonii*	Selenium	0.413	10	6	0.0001
2	Species *Parabacteroides goldsteinii*	Selenium	0.551	10	4	0.0001
3	Species *Bacteroides stercorirosoris*	Selenium	0.285	10	5	0.0001
4	Species Lachnospiraceae_bacterium_28_4	Selenium	−0.365	10	5	0.0099
5	Species *Lactobacillus reuteri*	Selenium	0.442	10	6	0.0135

### 4.3 Gene function and metabolic potential analysis

We used CARD annotation and found that 24 antibiotic resistance-associated genes were significantly different between the two groups (Supplementary Table S1). The results of the gene functional annotation of Egg-NOG revealed that 1,674 genes were significantly different between the two groups (Supplementary Table S2). Furthermore, a total of 37 functional COGs were significantly different between the selenium intervention group and the control group (Supplementary Table S3). A total of 21 significantly different functional genes associated with carbohydrate-active enzymes between the two groups were predicted (Supplementary Table S4). The two groups of genes were annotated by MvirDB, leading to the prediction of selenium effects on biological defense (Supplementary Table S5). PHI results revealed a total of 455 significant pathogen–host interactions (Supplementary Table S6). A total of 66 genes were significantly associated with cell communication and cell auto-induction by QS analysis (Supplementary Table S7). The TCDB results revealed a total of 834 genes associated with membrane transporters (Supplementary Table S8). VFDB analysis identified a total of 220 genes related to virulence factors that were significantly different after selenium intervention (Supplementary Table S9). We found that several pathways related to RNA anabolism were enriched after selenium intervention by KEGG pathway enrichment analysis; unfortunately, none of these pathways were significantly different (Supplementary Table S10). Based on the KEGG annotation results, the CMPs were calculated for the metabolites of the two groups of samples, and the differences in metabolic potential between the different groups were estimated, which showed that 48 metabolites including L-alanine, SO2, O2, d-biotin, L-asparagine, cephalin, and pyridoxine phosphate were significantly different (Supplementary Table S11).

## 5 Discussion

Breast cancer is the most common malignancy in women. Recent relevant studies have also illustrated that high-fat status is a high-risk factor for breast cancer development. Meanwhile, the content of cholesterol, low-density lipoprotein, etc. in the human body is regulated by dietary patterns, and the gut microbiota plays an important role in metabolic processes. Studies linking the gut microbiota to high-fat status breast cancer are increasing, with studies showing that alterations in BC gut microbiota in response to obesity and high-fat dietary intake are similar between humans and mice (Soto-Pantoja et al., 2021). Metagenomic sequencing and 16S rRNA gene sequencing are widely used in the analysis of the defined composition of microbial populations and can both be used to study the species composition of a community, the evolutionary relationships among species, and the diversity of the community. However, many of the sequences obtained from 16S rRNA gene sequencing are poorly annotated at the species level, while metagenomic sequencing based on 16S rRNA gene sequencing allows in-depth studies at the genetic and functional levels using GO, KEGG pathway, and other tools. At the same time, microbes can be identified at the species level (New and Brito, 2020). In this study, we attempted to explore the mechanism of selenium intervention in BC under high-fat diet conditions by examining the metabolic function of gut microbiota and related target genes in KEGG and COG pathways in BC-bearing mice on a high-fat diet after intervention with selenium.

Our results showed that the diversity of the gut microbiota BC-bearing mice under high-fat diet conditions after selenium intervention was significantly different under the premise that the sample biodiversity was sufficiently covered at the sequencing depth, and the distribution of the gut microbiota was also altered. The phyla represented by Bacteroidetes, Firmicutes, Deferribacteres, Spirochaetes, as well as the *Prevotella* sp. *MGM2, Muribaculum intestinale, Lactobacillus murinus*, and *Prevotella* sp. *MGM1* species were decreased in the gut microbiota in the selenium intervention group, whereas the Proteobacteria, Actinobacteria, and Verrucomicrobia phyla and the *Helicobacter ganmani, Helicobacter japonicus*, and *Akkermansia muciniphila* species were increased. The same results were shown in the LEfSe analysis. The analysis found no significant difference in enterotype and no significant difference in gene richness between the two groups, which revealed that selenium intervention did not significantly affect the gut microbiota genes and enterotype in BC-bearing mice on a high-fat diet. Studies have demonstrated that BC tumor growth can be inhibited by regulating the homeostasis of gut microbiota, producing short-chain fatty acids (SCFAs), and then interfering with the expression of tumor-associated proteins (Han et al., 2021). This was also confirmed by the elevated Firmicutes/Bacteroidetes (F/B) ratio in our study. Selenium can alter the production of medium and short-chain fatty acids, such as acetate and butyrate, which are essential for the maintenance of colonic mucosal integrity, thus regulating *Bacteroides* and *Prevotella* abundance (Christensen et al., 2018). Similar to the conclusion of Chi et al. (2022), the relative abundance of Lachnospiraceae and Prevotellaceae was increased and the abundance of Helicobacteraceae was decreased after increasing the content of selenium in the ingested food. Furthermore, the correlation analysis suggested that selenium could optimize the functional network in the gut microbiota and optimize the interaction between the gut microbiota and host. It has been reported that the supernatant of *Faecalibacterium prausnitzii* can inhibit the growth of BC cells by inhibiting the IL-6/STAT3 pathway (Ma et al., 2020), suggesting that this class of bacteria may contribute to BC prevention, while reduction of this class of bacteria may promote BC progression. In addition, our previous study found that decreasing STAT3 phosphorylation could effectively induce apoptosis and pyroptosis in BC cells exposed to high-fat conditions (Liu M. et al., 2022). There was a trend toward enrichment in several KEGG pathways related to RNA anabolism in the selenium intervention group, although not significant, and notably, there was a trend toward decreased fatty acid biosynthesis related to fatty acid anabolism, suggesting a possible role for selenium in the biosynthesis of fatty acids in BC-bearing mice fed with a high-fat diet. Selenium can inhibit fat accumulation and has anti-inflammatory activity which has been confirmed by the study of Liu G. et al. (2022). Our results annotated by CAZy also confirm that selenium can interfere with carbohydrate-active enzymes in BC-bearing mice fed a high-fat diet, which is produced by intervening with the gut microbiota of limited lipogenesis. Selenium also exerts an effect on antibiotic resistance, and the Se NP-ε-PL generated by Huang et al. (2020) showed good antibacterial activity against eight different bacteria, including partially drug-resistant strains. Based on the results of CARD annotation, we found that selenium could modulate antibiotic resistance in high-fat status BC tumor-bearing mice by intervening with the gut microbiota function. Furthermore, Egg-NOG analysis results showed that selenium could have an effect on pathogen biological attack factors, cell auto-induction, and membrane transporters by intervening gut microbiota. Prediction of the metabolic potential of the samples from the two groups revealed that selenium intervention may improve the metabolic capacity of BC-bearing mice on a high-fat diet to L-alanine, sulfur dioxide, oxygen, biotin, and L-asparagine, in which biotin plays an important role in biochemical pathways, such as fat synthesis and gluconeogenesis. The combination of biotin and prebiotic supplementation may help prevent the deterioration of metabolic status in severely obese patients (Belda et al., 2022).

Investigating the intervention effects of organic selenium on gut microbiota dysbiosis in BC may contribute to our understanding of this disease’s etiology and pathomechanisms and facilitate targeted new prediction and treatment. Potential limitations of the present study are as follows: (1) The gut epithelium and fecal supernatant were helpful for further studies, but related samples were not collected. (2) The sample marker gene database used for analyzing the gut microbiota, genetic functions, and related metabolites in mice using metagenomic sequencing may have included most species and strain level information rather than 100% of all existing species or organisms. (3) Metabolomics was not utilized when performing microbial metabolite analysis, but rather samples were analyzed in terms of DNA and gut microbiota abundance. The actual microbial metabolite profile may differ from metabolic potential analysis during DNA transcription and translation. (4) The experimental model mice and the 4 T1 breast cancer tumor cell line used for research have shed new light on BC research, but this does not represent the actual situation of the gut microbiota in all high-fat state BC patients in the clinic.

High-fat microenvironment is not only a high-risk factor for breast cancer but also has a negative impact on breast cancer. This study linked breast cancer with obesity by constructing a high-fat breast cancer-bearing mouse model. We first observed changes in the composition, gene function, and homeostasis of the gut microbiota by intervening in the gut microbiota under this environment with trace elements. This sheds light on the idea that breast cancer patients who are high in fat may have an improved gut microbiota through adequate selenium supplementation. Selenium will benefit the gut microbiota metabolism of patients, promote the growth of probiotics, play a role in improving inflammation and other effects, while inhibiting harmful bacteria, and play a positive role in the treatment of breast cancer. At the same time, we also need to realize that mice experiments are not entirely identical to those in humans. Mice and humans have significant differences in size and physiological structure, and the structure and functions of their organs vary. These factors may lead to different effects on experimental results. The advantage of animal experiments lies in their reproducibility and controllability. Therefore, we suggest that the results of this study are reliable, confirming the positive role of selenium in the treatment of breast cancer, with certain clinical significance.

## 6 Conclusion

After selenium intervention, Proteobacteria, Actinobacteria, Verrucomicrobia, and other bacteria were increased and Bacteroidetes, Firmicutes, Spirochaetes, and other bacteria were decreased in breast cancer mice with a high-fat diet. This suggests that selenium can influence the gut microbiota by regulating related gene functions. It adjusts the microbial community’s stability in terms of species diversity and abundance. This mechanism may involve altering the synthesis of fatty acids and modulating the expression of genes related to tumor-associated proteins. In other words, selenium appears to regulate the balance of different bacterial populations in the gut by targeting specific genes in those bacterial groups. Its action might involve correcting any disruptions caused by the high-fat diet, potentially by altering the metabolic pathways contributing to fatty acid production. This, in turn, could have an indirect effect on the regulation of genes involved in tumor development, resulting in a more stable and potentially healthier gut ecosystem. Further research is needed to confirm these mechanisms.

Intervention with gut microbiota function using selenium may provide a new prospect in the treatment of high-fat status BC. The results of this study are validated and contribute to the development of novel predictive and therapeutic approaches for BC under high-fat diet conditions in future.

## Data availability statement

The datasets presented in this study can be found in online repositories. The names of the repository/repositories and accession number(s) can be found at: https://www.ncbi.nlm.nih.gov/, sra/PRJNA857801.

## Ethics statement

The animal study was approved by Ethics board of Beijing Viewsolid Biotechnology Co., Ltd. (No. 202000038). The study was conducted in accordance with the local legislation and institutional requirements.

## Author contributions

YL: Conceptualization, Data curation, Formal analysis, Funding acquisition, Investigation, Methodology, Project administration, Resources, Software, Supervision, Validation, Visualization, Writing – original draft, Writing – review & editing. ML: Conceptualization, Formal analysis, Methodology, Project administration, Software, Writing – original draft. BK: Conceptualization, Data curation, Investigation, Supervision, Visualization, Writing – review & editing. GZ: Investigation, Methodology, Resources, Supervision, Validation, Visualization, Writing – review & editing. QZ: Funding acquisition, Investigation, Methodology, Project administration, Resources, Supervision, Validation, Visualization, Writing – review & editing.
