# Supplementary material for: The role of selenium intervention in gut microbiota homeostasis and gene function in mice with breast cancer on a high-fat diet
Source: Front Microbiol. 2024 Jul 31;15:1439652. doi: 10.3389/fmicb.2024.1439652 (PMC11322145; doi:10.3389/fmicb.2024.1439652)
Supplement: Supplementary file 1 [file Table_1.docx]

TABLE S1: Test results for significance of differences between groups were annotated with EggNog(top10)

|  | feature | Control | Selenium | W | pvalue |
| --- | --- | --- | --- | --- | --- |
| 1 | CP_0488 | 0 | 1.738 | 0 | 0.006 |
| 2 | M1-580 | 0 | 2.060 | 0 | 0.006 |
| 3 | MA20_23860 | 0 | 2.225 | 0 | 0.006 |
| 4 | MA20_27595 | 0 | 1.856 | 0 | 0.006 |
| 5 | MA20_28320 | 0 | 1.635 | 0 | 0.006 |
| 6 | PMM | 0 | 0.861 | 0 | 0.006 |
| 7 | XK27_02315 | 0 | 2.838 | 0 | 0.006 |
| 8 | XK27_04590 | 0 | 6.493 | 0 | 0.006 |
| 9 | XK27_05000 | 0 | 1.325 | 0 | 0.006 |
| 10 | bCE_4747 | 0 | 17.934 | 0 | 0.006 |

Table S2 Results of intergroup comparisons of card annotated outcomes. (top20)

|  | feature | Control | Selenium | W | pvalue |
| --- | --- | --- | --- | --- | --- |
| 1 | gimA family macrolide glycosyltransferase | 0 | 11.031 | 0 | 0.006 |
| 2 | AAC(3) | 23.353 | 5.797 | 24.000 | 0.010 |
| 3 | DHA beta-lactamase | 28.560 | 1.248 | 24.000 | 0.010 |
| 4 | SPG beta-lacatamase | 11.169 | 0.315 | 24.000 | 0.010 |
| 5 | TLA beta-lactamase | 13.349 | 0.753 | 24.000 | 0.010 |
| 6 | subclass B3 PEDO beta-lactamase | 16.583 | 0.610 | 24.000 | 0.010 |
| 7 | van ligase | 7.580 | 17.618 | 0 | 0.010 |
| 8 | CepS beta-lactamase | 2.778 | 0.123 | 24.000 | 0.014 |
| 9 | tetracycline inactivation enzyme | 2.476 | 14.092 | 1.000 | 0.019 |
| 10 | vanR | 7.227 | 1.599 | 23.000 | 0.019 |
| 11 | vanS | 6.847 | 0.521 | 23.000 | 0.025 |
| 12 | aminocoumarin resistant parY | 0 | 0.061 | 3.000 | 0.026 |
| 13 | aminocoumarin self resistant parY | 0 | 0.061 | 3.000 | 0.026 |
| 14 | OCH beta-lactamase | 5.100 | 0 | 22.000 | 0.031 |
| 15 | subclass B1 Vibrio cholerae varG beta-lactamase | 0.421 | 3.317 | 2.000 | 0.031 |
| 16 | AAC(6') | 8.843 | 2.099 | 22.000 | 0.038 |
| 17 | resistance-nodulation-cell division (RND) antibiotic | 7.198 | 1.217 | 22.000 | 0.038 |
| 18 | OKP beta-lactamase | 2.378 | 14.226 | 2.000 | 0.038 |
| 19 | ole glycosyltransferase | 8.766 | 27.710 | 2.000 | 0.038 |
| 20 | aminocoumarin self resistant parY | 0.461 | 2.500 | 2.000 | 0.040 |

TABLE S3: Results between groups comparisons of COG annotations(top 5)

|  |  | HFD-g2 | HFD-g6 | W | pvalue |
| --- | --- | --- | --- | --- | --- |
| 1 | EHJ | 0 | 2.801 | 0 | 0.006 |
| 2 | E | 31996.080 | 36302.436 | 0 | 0.010 |
| 3 | FG | 296.727 | 409.194 | 0 | 0.010 |
| 4 | FP | 144.546 | 236.673 | 0 | 0.010 |
| 5 | GJM | 14.168 | 37.031 | 0 | 0.010 |

TABLE S4: Results between groups comparisons of CAZy annotations（top 5）

|  |  | Control | Selenium | W | pvalue |
| --- | --- | --- | --- | --- | --- |
| 1 | GH101 | 83.461 | 183.448 | 0 | 0.010 |
| 2 | GH102 | 1.325 | 10.874 | 0 | 0.010 |
| 3 | GH15 | 9.584 | 23.914 | 0 | 0.010 |
| 4 | GH37 | 5.236 | 14.655 | 0 | 0.010 |
| 5 | GH73 | 2.237 | 20.446 | 0 | 0.010 |

Table S5 Results between groups comparisons of MvirDB annotations (top10)

|  |  |  | Control | Selenium | W | pvalue |
| --- | --- | --- | --- | --- | --- | --- |
| 1 | AcrA_protein |  | 0 | 4.125 | 0 | 0.006 |
| 2 | BexD` |  | 0 | 0.579 | 0 | 0.006 |
| 3 | CP4-6_prophage;_DNA-binding_protein |  | 0 | 3.866 | 0 | 0.006 |
| 4 | HCNC |  | 0 | 3.311 | 0 | 0.006 |
| 5 | IS3_element_protein_InsE |  | 0 | 3.900 | 0 | 0.006 |
| 6 | RecName:Full=Casein_kinase_II_subunit_alpha;_AltName… |  | 0 | 1.587 | 0 | 0.006 |
| 7 | SubName:Full=73;_SubName:_Full=Immediate-early_protein;_ |  | 0 | 6.167 | 0 | 0.006 |
| 8 | SubName:Full=EBNA-3A;_ |  | 0 | 8.888 | 0 | 0.006 |
| 9 | SubName:Full=Pertactin;_ |  | 0 | 1.729 | 0 | 0.006 |
| 10 | bacitracin_resistance_protein |  | 0 | 2.053 | 0 | 0.006 |

TABLE S6: Results between groups comparisons of PHI annotations（top 10）

|  |  | Control | Selenium | W | pvalue |
| --- | --- | --- | --- | --- | --- |
| 1 | G4MZ47 | 0 | 1.506 | 0 | 0.006 |
| 2 | I1RAY2 | 0 | 1.682 | 0 | 0.006 |
| 3 | Q03023 | 0 | 4.018 | 0 | 0.006 |
| 4 | Q04LP0 | 0 | 1.141 | 0 | 0.006 |
| 5 | Q63K73 | 0 | 6.352 | 0 | 0.006 |
| 6 | P87198 | 0.021 | 10.028 | 0 | 0.009 |
| 7 | Q2YIT5 | 0.087 | 2.973 | 0 | 0.009 |
| 8 | Q70Q35 | 0.057 | 2.369 | 0 | 0.009 |
| 9 | Q8YFR7 | 0.147 | 3.892 | 0 | 0.009 |
| 10 | Q9C0N4 | 0.087 | 7.158 | 0 | 0.009 |

TABLE S7: Results between groups comparisons of QS annotations（top 10）

|  |  | Control | Selenium | W | pvalue |
| --- | --- | --- | --- | --- | --- |
| 1 | A0A1C6EWK9 | 0 | 4.673 | 0 | 0.006 |
| 2 | A0A255S7Y1 | 0 | 8.522 | 0 | 0.006 |
| 3 | P55629 | 0.065 | 1.933 | 0 | 0.009 |
| 4 | A0A0E0V4N7 | 15.910 | 0.577 | 24 | 0.010 |
| 5 | A0A0H3FDM2 | 3.554 | 10.466 | 0 | 0.010 |
| 6 | A0A1B1KN52 | 1.043 | 7.719 | 0 | 0.010 |
| 7 | A0A1B8HRY3 | 6.733 | 0.716 | 24 | 0.010 |
| 8 | A0A1M0D247 | 1.730 | 16.789 | 0 | 0.010 |
| 9 | A0A1X9TE10 | 0.925 | 8.624 | 0 | 0.010 |
| 10 | A0A2S4QJN3 | 14.370 | 0.372 | 24 | 0.010 |

TABLE S8: Results between groups comparisons of TCDB annotations（top 10）

|  |  | Control | Selenium | W | pvalue |
| --- | --- | --- | --- | --- | --- |
| 1 | 1.A.1.14.6 | 0 | 0.784 | 0 | 0.006 |
| 2 | 1.A.13.1.7 | 0 | 10.025 | 0 | 0.006 |
| 3 | 1.A.77.2.14 | 0 | 3.219 | 0 | 0.006 |
| 4 | 1.B.12.13.2 | 0 | 3.216 | 0 | 0.006 |
| 5 | 1.B.12.2.7 | 0 | 2.132 | 0 | 0.006 |
| 6 | 1.B.12.5.7 | 0 | 17.148 | 0 | 0.006 |
| 7 | 1.B.12.8.1 | 0 | 4.651 | 0 | 0.006 |
| 8 | 1.B.16.2.2 | 0 | 7.927 | 0 | 0.006 |
| 9 | 1.B.20.3.7 | 0 | 15.605 | 0 | 0.006 |
| 10 | 1.B.92.1.1 | 0 | 1.960 | 0 | 0.006 |

TABLE S9: Results between groups comparisons of VFDB annotations（top 10）

|  |  | Control | Selenium | W | pvalue |
| --- | --- | --- | --- | --- | --- |
| 1 | AI108 | 0 | 2.272 | 0 | 0.006 |
| 2 | AI446 | 0 | 7.770 | 0 | 0.006 |
| 3 | SS106 | 0 | 2.132 | 0 | 0.006 |
| 4 | SS135 | 0 | 5.889 | 0 | 0.006 |
| 5 | SS165 | 0 | 2.181 | 0 | 0.006 |
| 6 | TX319 | 0 | 6.022 | 0 | 0.006 |
| 7 | VF0426 | 0 | 2.847 | 0 | 0.006 |
| 8 | AI339 | 0.031 | 6.241 | 0 | 0.009 |
| 9 | CVF243 | 0.095 | 11.521 | 0 | 0.009 |
| 10 | AI100 | 14.181 | 0.670 | 24 | 0.010 |

Table S10 Based on the differential results from Ko, enrichment analysis was used to obtain the KEGG pathways that were significantly different among the different groups (top 10).

|  | pathway | p.geomean | stat.mean | p.val | set.size | Selenium |
| --- | --- | --- | --- | --- | --- | --- |
| 1 | ko03018.RNA.degradation | 0.062 | 1.575 | 0.062 | 22 | up |
| 2 | ko00970.Aminoacyl.tRNA.biosynthesis | 0.081 | 1.424 | 0.081 | 31 | up |
| 3 | ko00195.Photosynthesis | 0.093 | 1.346 | 0.093 | 22 | up |
| 4 | ko00061.Fatty.acid.biosynthesis | 0.094 | -1.349 | 0.094 | 17 | down |
| 5 | ko00240.Pyrimidine.metabolism | 0.095 | 1.318 | 0.095 | 73 | up |
| 6 | ko03030.DNA.replication | 0.098 | -1.316 | 0.098 | 26 | down |
| 7 | ko00670.One.carbon.pool.by.folate | 0.116 | 1.231 | 0.116 | 17 | up |
| 8 | ko03020.RNA.polymerase | 0.117 | 1.228 | 0.117 | 11 | up |
| 9 | ko00190.Oxidative.phosphorylation | 0.124 | 1.163 | 0.124 | 72 | up |
| 10 | ko00770.Pantothenate.and.CoA.biosynthesis | 0.127 | 1.163 | 0.127 | 22 | up |

Table S11 CMPs were calculated using the PRMT method, and analysis of differences between groups revealed several significant differences in metabolic potential (top10).

|  |  | Metabolite | Control | Selenium | W | pvalue |
| --- | --- | --- | --- | --- | --- | --- |
| 1 | C00041 | L-Alanine | 0 | 215,078.68 | 0 | 0.006 |
| 2 | C09306 | Sulfur dioxide | 0 | 215,078.68 | 0 | 0.006 |
| 3 | C00007 | Oxygen | 115,274.72 | 601,031.50 | 0 | 0.010 |
| 4 | C00120 | Biotin | 314,575.83 | 637,720.75 | 0 | 0.010 |
| 5 | C00152 | L-Asparagine | -119,335.17 | 1,066,719.55 | 0 | 0.010 |
| 6 | C00350 | Phosphatidylethanolamine | 10,649.08 | 541,085.00 | 0 | 0.010 |
| 7 | C00627 | Pyridoxine phosphate | 215,444.17 | 625,641.00 | 0 | 0.010 |
| 8 | C02972 | Dihydrolipoylprotein | 125,261.67 | 693,816.00 | 0 | 0.010 |
| 9 | C04631 | UDP-N-acetyl-3-(1-carboxyvinyl)-D- | -64,879.00 | 688,963.75 | 0 | 0.010 |
| 10 | C04851 | MurAc-diphospho-undecaprenol | -18,425.33 | 726,519.50 | 0 | 0.010 |
